# Completely non-invasive prediction of IDH mutation status based on preoperative native CT images

**DOI:** 10.1038/s41598-024-77789-6

**Published:** 2024-11-05

**Authors:** Manfred Musigmann, Melike Bilgin, Sabriye Sennur Bilgin, Hermann Krähling, Walter Heindel, Manoj Mannil

**Affiliations:** University Clinic for Radiology, University Münster and University Hospital Münster, Albert- Schweitzer-Campus 1, 48149 Münster, Germany

**Keywords:** Glioma, IDH mutation status, Machine learning, Artificial intelligence, Neuroimaging, Computed tomography (CT), Radiomics, Diagnostic markers, Neoplasm staging

## Abstract

The isocitrate dehydrogenase (IDH) mutation status is one of the most important markers according to the 2021 WHO classification of CNS tumors. Preoperatively, this information is usually obtained based on invasive biopsies, contrast-enhanced MR images or PET images generated using radioactive tracers. However, the completely non-invasive determination of IDH mutation status using routinely acquired preoperative native CT images has hardly been investigated to date. In our study, we show that radiomics-based machine learning allows to determine IDH mutation status based on preoperative native CT images both with very high accuracy and completely non-invasively. Based on independent test data, we are able to correctly identify 91.1% of cases with an IDH mutation. Our final model, containing only six features, exhibits a high area under the curve of 0.847 and an excellent area under the precision-recall curve of 0.945. In the future, such models may be used for a completely non-invasive prediction of important genetic markers, potentially allowing treating physicians to reduce the number of biopsies and speed up further treatment planning.

## Introduction

Gliomas are the most common primary brain tumors and account for approximately half of all intracranial tumors^1,2^. In the United States, six cases per 100,000 people are diagnosed each year^3^. Most gliomas are diffuse gliomas that infiltrate the parenchyma of the central nervous system (CNS) extensively. The precise classification of gliomas is of fundamental importance for the prognosis and further treatment of the affected patients^4^. The current version of the WHO classification of CNS tumors has been published in 2021^5–7^. A seven-stage classification scheme is now used to characterize gliomas^4^. Since the last versions of the WHO classification, tumors have been subdivided into more molecularly and biologically defined entities. The 2021 WHO classification primarily differentiates adult-type diffuse gliomas into the three main tumor types “astrocytoma, IDH-mutant”, “oligodendroglioma, IDH-mutant and 1p/19q-codeleted” and “glioblastoma, IDH-wildtype”. The first three steps of the seven-stage differentiation scheme are particularly important for the differentiation between these specifications. In these three steps, mutations in isocitrate dehydrogenase (IDH) genes IDH1 and IDH2, alpha-thalassemia/mental retardation syndrome X-linked (ATRX) expression (nuclear ATRX is retained or lost) and possible co-deletions of 1p/19q (deletion of the short arm of chromosome 1 and the long arm of chromosome 19) are evaluated. The ATRX gene is essential for the chromatin remodeling and maintenance of genomic stability and is particularly important in diffuse gliomas of the adult type^8–10^. 1p/19q codeletions, on the other hand, play a crucial role in the tumor prognosis of diffuse low-grade gliomas and are important for the classification of oligodendrogliomas^11^. However, in particular the first step of the above-mentioned seven-step scheme, i.e. the prediction of the IDH mutation status, is of fundamental importance for further therapy planning in the context of personalized medicine. Tumors without IDH mutation (IDH-wildtype tumors) are classified with the highest WHO grade 4, while IDH-mutated tumors usually have a WHO grade of 2 or 3. The distinction between WHO grades 2 and 3 is primarily based on mitotic activity; however, this differentiation has become less significant compared to previous versions of the WHO classification of CNS tumors.

The treatment of gliomas differs greatly depending on the tumor subtype respectively the WHO grade. The standard treatment for lower-grade gliomas is maximal safe resection followed by surveillance in low-risk patients and radiation and adjuvant chemotherapy in high-risk patients^12–14^. The standard of care for higher-grade gliomas includes maximum feasible resection or biopsy, followed by radiation, stereotactic radiosurgery, and adjuvant chemotherapy (such as temozolomide)^15–18^.

In addition to the subtype of the tumor, the expected survival time depends strongly on the age of the patient^19^. IDH-mutated gliomas usually have a better prognosis with a median survival time of over 12 years^20^, but they often show a malignant transformation towards higher WHO grades as the disease progresses. IDH wildtype gliomas are mostly glioblastomas (GBM), which usually exhibit a very aggressive behavior. Despite multimodal treatment, the average survival time of affected patients is only 12 to 15 months^21,22^.

Due to the reasons mentioned above, the reliable and rapid determination of the IDH mutation status is extremely important for the further treatment planning. The enzymatic activity of the proteins that are produced from normal and mutant IDH1 and IDH2 genes can be determined, for example, in cultured glioma cells that are transfected with these genes^23^. However, these and other comparable methods for the determination of the IDH mutation status are invasive, often time-consuming and require appropriate laboratory capacity. Accordingly, a non-invasive, rapid, and reliable method to determine the IDH mutation status would have considerable advantages. One way to determine such molecular markers non-invasively is to combine radiomics with artificial intelligence methods, or more precisely, machine learning. Radiomics is still a relatively young branch of medical imaging. Quantitative features/information are extracted from medical images, such as MR (magnetic resonance) images, which are subsequently used for diagnostic purposes^24,25^. The large number of radiomic features extracted from the medical images are usually composed of morphological/shape, statistical and textural features. An overview of radiomic features, their description and meaning can be found, for example, in van Griethuysen et al.^26^.

Machine learning combined with radiomics has already demonstrated its enormous potential in numerous medical studies and applications in recent years. To give a few examples, Ari et al. have shown that radiomics-based machine learning is a promising tool to predict the occurrence of pseudoprogression in high-grade gliomas, thus potentially allowing physicians to reduce the use of biopsies and invasive histopathology^27^. Machine learning algorithms can also be used to make accurate predictions about whether or not a meningioma can be completely resected by surgery^28^. A number of studies have investigated the differentiability of gliomas of different WHO grades using MR image-based machine learning^29,30^. Specifically, the prediction of the IDH mutation status based on MR images has also already been analyzed in several studies^31–36^. Kasap et al. investigated which of the commonly used MRI sequences is particularly suitable for the radiomics-based prediction of IDH mutation status^37^. The determination of IDH and ATRX mutations using automated machine learning (AutoML) has also already been investigated^38^. In contrast to using MR images acquired with the conventional or advanced MRI sequences, some studies also attempt to determine IDH mutation status based on positron emission tomography (PET) images^39–41^.

However, all the studies mentioned for the determination of IDH mutation status are based on MR images or, in some cases, on PET images. The question arises to what extent computed tomography (CT) images are also suitable for a radiomic-based, non-invasive prediction of the IDH mutation status. It is conceivable, for example, that MR images are not (yet) available for a patient, but CT images have already been acquired. Irrespective of whether MR images are available, the question also arises whether CT images pertains valuable information which is equally or perhaps even better suited for the non-invasive determination of IDH mutation status.

These questions are the aim of our study. Using preoperative native CT images, we investigate to what extent they are suitable for a completely non-invasive prediction of the IDH mutation status using radiomics-based machine learning. Two special features of our study are therefore that we determine the IDH mutation status based on CT images and that the medical images used for this purpose were obtained both without the administration of a contrast agent and without the administration of a radioactive tracer (i.e., based on native CT images). In our study, we are also interested in the predictability of reclassifications to be made based on the current 2021 WHO classification. With regard to our study, the main difference between the 2016 and 2021 WHO classifications is that IDH-wildtype gliomas were assigned to WHO grades II, III and IV according to the 2016 WHO classification (grade II diffuse astrocytoma, grade III anaplastic astrocytoma, grade IV glioblastoma). According to the 2021 WHO classification, however, IDH-wildtype gliomas are assigned to WHO grade 4 without exception. Therefore, the WHO classification of 2021 results in a reclassification of all IDH-wildtype gliomas of grades II and III. However, this does not apply to WHO grade IV IDH-wildtype tumors. For this reason, we only included patients with grade II or III gliomas according to the 2016 WHO classification in our study cohort and explicitly did not include WHO grade IV patients. Using radiomics-based machine learning to determine IDH mutation status in this study, we can therefore also make a statement about which patients need to be reclassified according to the current WHO classification, i.e., are now assigned to WHO grade 4.

## Materials and methods

Our study was performed in compliance with the Declaration of Helsinki and approved by the local ethics committee (Ärztekammer Westfalen Lippe and University of Münster, 2021-596-f-S). Due to its retrospective nature, written informed consent was waived by the local ethics committee (Ärztekammer Westfalen Lippe and University of Münster). As explained above, the aim of our study is to predict IDH mutation status in gliomas using radiomics and machine learning based on preoperative native CT images. Therefore, we retrospectively searched the Picture Archiving and Communication System (PACS) of our hospital for patients with gliomas diagnosed between 2008 and 2020. Most common symptoms in patients with gliomas at initial diagnosis are found to be epileptic seizures and headaches^42,43^. The first clinical evaluation of our patients consisted of undergoing a thorough neurological examination, following a diagnostic imaging method such as a computed tomography or magnetic resonance imaging to detect a primary brain tumor. For the final histopathological diagnosis of a glioma all of our patients underwent surgery with biopsy results confirming a glioma according to the European Association of Neuro-Oncology (EANO) guidelines^4^.

To be able to analyze possible reclassifications to WHO grade 4 due to the 2021 WHO classification of CNS tumors, we did not include grade IV gliomas according to the 2016 WHO classification. Preoperative native CT images of patients with histopathological confirmed WHO grade II and III gliomas and known IDH mutation status were available for 92 patients. In summary, all patients were included in our study for whom the following four criteria applied simultaneously: (a) diagnosis between 2008 and 2020, (b) histopathological confirmed grade II or III glioma according to the WHO classification of 2016, (c) known IDH mutation status and (d) existing preoperative native CT images. None of the patients who fulfilled all of these four criteria simultaneously were excluded from the study (e.g. due to poor image quality or other possible exclusion criteria). Our final study cohort of 92 patients consists of 39 female/53 male, or 70 IDH-mutated and 22 IDH-wildtype cases. The 22 IDH-wildtype cases in turn consist of 12 cases with WHO grade II and 10 cases with WHO grade III. These 22 cases are now all assigned to WHO grade 4 according to the 2021 WHO classification, i.e. reclassified. The demographic and histopathological characteristics of our study are summarized in Table 1. At this point, it should be noted that in accordance with the WHO standard, we use in our study Roman numbers (i.e., grade I to IV) according to the 2016 WHO classification of CNS tumors and Arabic numbers (i.e. grade 1 to 4) according to the 2021 WHO classification of CNS tumors.

**Table 1:** Demographic and histopathological characteristics of the study cohort used to determine IDH mutation status.

	Training data	Independent test data	Total data
Number of patients	74	18	92
Gender (in %)			
Female	42.32	42.67	42.39
Male	57.68	57.33	57.61
Mean age (in years)	45.95	46.04	45.97
IDH status (in %)			
Mutant	75.68	77.78	76.09
Wildtype	24.32	22.22	23.91

### Image acquisition and radiomics

**Fig. 1:**
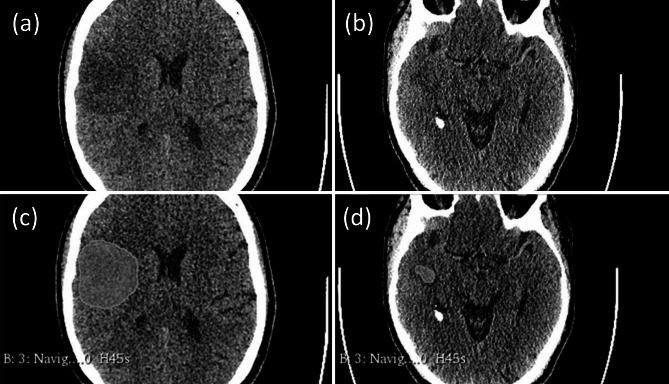
Preoperative native CT images (axial slice) of two patients with **IDH-mutated** (left images) and non-IDH-mutated (**IDH wildtype**) glioma (right images). Figures (a) and (b) show the CT images without the overlaid segmented areas. The areas marked in green in figures (c) and (d) show the corresponding semi-automatic segmentations with 3D Slicer. Left figures (a) + (c): Case with IDH mutation. 53-year-old patient with an IDH mutated astrocytoma located in the right insular region. Right figures (b) + (d): Case without IDH mutation. 59-year-old patient with a IDH wildtype glioma located in the right temporal lobe.

The CT-images used for our study were obtained with dual energy CT scanners Somatom Force and Somatom Definition AS from Siemens Healthineers, Germany. We used preoperative native computed tomography imaging for navigation with a typical voxel size of 512 × 512 and a slice thickness of 1 mm (Navigation CT 1.0 mm H45s). We downloaded navigation CT images in DICOM format and pseudonymized the DICOM header. Segmentation of the hypodense tumor parts on the preoperative native CT images (CT images without contrast enhancement) was semi-automatically performed using the 3D Slicer open-source software platform (version 4.10, www.slicer.org) and utilizing the Segmentation Wizard plugin by two readers. Gliomas commonly present as hypodense lesions on native CT images. The low CT density of the tumor using Hounsfield units was used to differentiate tumor tissue from normal brain parenchyma. The segmentation process for all imaging was performed and reviewed by board-certified neuroradiologists. Expert consensus was used in anatomically difficult segmentation. Figure 1 shows two examples of the semi-automatic segmentation with 3D Slicer. The two images on the left show a preoperative native CT image of a patient with an IDH-mutated astrocytoma and the two images on the right show a corresponding CT image of a patient with an IDH-wildtype glioma (without IDH mutation). The areas marked in green in the images below (c) and (d) show the semi-automatic segmentation with 3D Slicer.

The radiomics features were calculated using the open-source PyRadiomics software (version v3.0.1, https://pyradiomics.readthedocs.io/en/latest/), which is available as an implementable plugin for the 3D slicer platform. The radiomic features were extracted by hand-delineated regions of interest (ROI) from the CT images of each patient. In detail, the total of 107 features consist of 18 first order statistics features, 14 shape-based features, 24 Gy level co-occurrence matrix features, 16 Gy level run length matrix features, 16 Gy level size zone matrix features, 5 neighbouring gray tone difference matrix features, and 14 Gy level dependence matrix features. In addition, our database contained the gender and age of the patients. All features were z-score transformed and subjected to a 95% correlation filter to account for possible redundancy between the features. The datasets used and/or analysed during the current study are available from the corresponding author on reasonable request.

### Statistical analysis

**Fig. 2:**
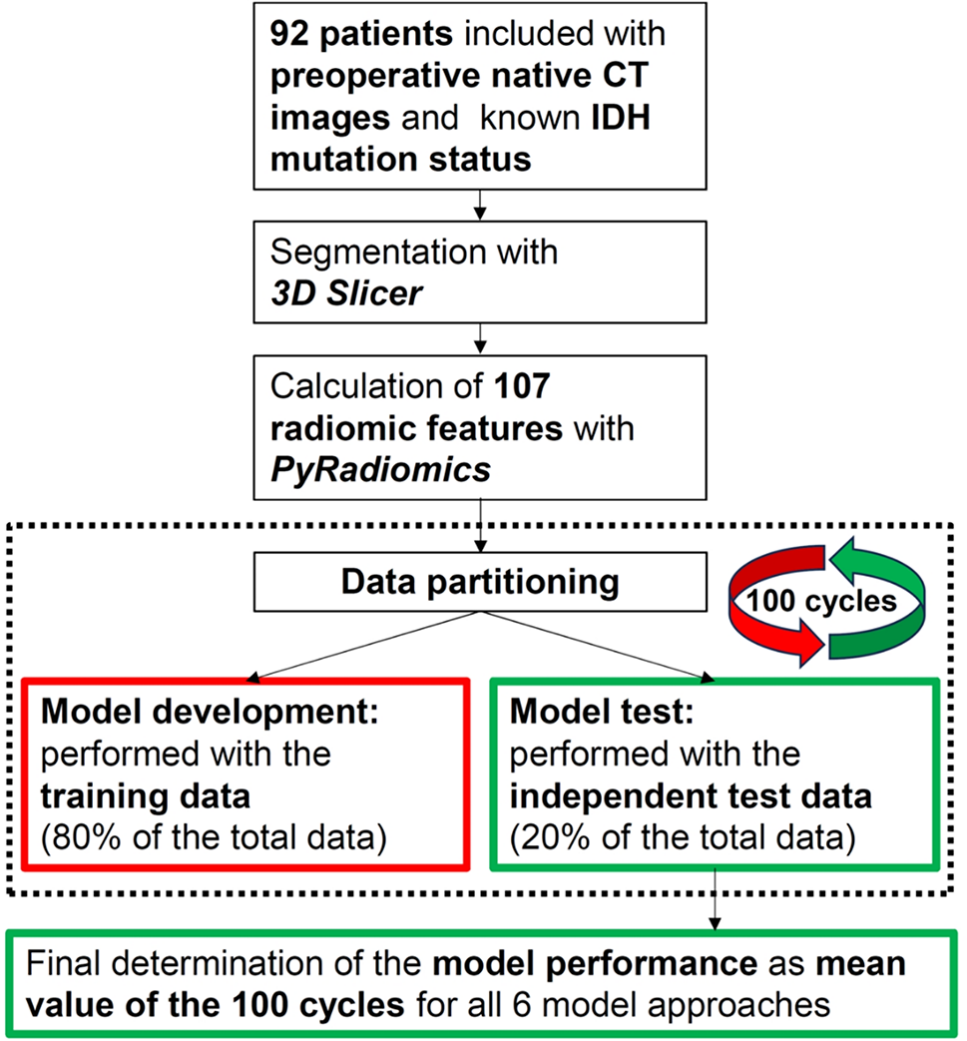
Flowchart describing the methodological approach. The study cohort for the prediction of IDH mutation status based on preoperative native CT images consists of 92 patients. Features were preselected using recursive feature elimination. Six different machine learning algorithms were tested. Each of these six models was developed 100 times with new data partitioning each time and tested with independent test data afterwards. The final model performance was determined as the average value of the 100 runs.

Statistical analysis was performed using R software (version 4.1.2, https://cran.r-project.org/bin/windows/base/old/4.1.2/). The 92 patients included in our study cohort were randomly assigned to training data and independent test data. We used a stratified 4:1 ratio (training data/ independent test data) with a balanced distribution of IDH-mutant and IDH-wildtype cases (see Table 1) between the two samples. The training data were used for feature preselection and subsequent model developments, including 10-fold cross-validation to optimize hyperparameters included in the models. The performance achieved with each final model was determined based on independent test data. It is important to note that we performed the division into training and test data, the model development, and the subsequent model testing 100 times for each individual model. Thus, we used 100 different sets of training data and 100 different sets of test data to eliminate random effects associated with the data partitioning as much as possible. The entire procedure is described in detail in Musigmann et al.^44^. For better understanding, we have also illustrated the procedure used in a flow chart (see Fig. 2).

Feature preselection was performed using recursive feature elimination (RFE) (see, for example, Darst et al.^45^) based on the random forest algorithm. For each of the 100 different training data sets, we first used RFE to preselect features for the subsequent model constructions. In the subsequent model developments, five different conventional machine learning algorithms and a neural network were trained and tested. In detail, we tested linear discriminant analysis (LDA), Lasso (least absolute shrinkage and selection operator) regression, the random forest algorithm, a gradient boosting machine (GBM), XGBoost and a neural network to predict the IDH mutation status. All hyperparameters included in the algorithms were determined using 10-fold cross-validation. The models were optimized by maximizing the area under the curve (AUC) of the receiver operator characteristic (ROC). Finally, the best of the 6 models/approaches with the highest performance was determined.

Without exception, all performance values were calculated as averages of 100 cycles (i.e., using 100 different data splits into training data and test data) based on the respective independent (unseen) test data. Given the imbalance in our class distribution of 3.16:1 (IDH-mutant vs. IDH-wildtype cases) in our data (compare Table 1), we used performance metrics that are particularly suitable for such imbalances. In detail, the predictive power of the individual models was analyzed using AUC, accuracy, Cohen’s kappa, precision, recall, AUPRC and F1-score. Cohen’s kappa is defined as: (observed accuracy – expected accuracy) / (1- expected accuracy)). Cohen’s kappa provides a more objective description of model performance than accuracy in case of imbalanced data. Higher, i.e., better values (closer to the + 1 value) for Cohen’s kappa are much more difficult to achieve for unbalanced class distributions. The opposite is true for accuracy. The precision is equivalent to the positive predictive value (PPV) and describes the proportion of correctly predicted cases with IDH mutation in relation to all predicted cases with IDH mutation. Recall describes the proportion of correctly predicted cases with an IDH mutation in relation to the real (true) number of cases with an IDH mutation and corresponds to the sensitivity. AUPRC is the area under the precision-recall curve. Finally, the F1-Score is defined as (2 × precision × recall) / (precision + recall).

## Results

As mentioned above, we used recursive feature elimination for feature preselection, followed by five conventional machine learning algorithms and a neural network for subsequent model constructions. Table 2 summarizes the performance values achieved with these six different machine learning algorithms. The table shows the mean performance values averaged over 100 cycles. All performance values were calculated using independent test data. The values in brackets represent the 95% confidence interval. The six different machine learning algorithms result in relatively similar, high mean discriminatory power values. Overall, Lasso regression yields the best performance in predicting the IDH mutation status. The Lasso regression results in a mean AUC of 0.807 and an excellent mean AUPRC of 0.925. Using this approach, an average of 91.1% of the IDH mutations are correctly detected (Recall/Sensitivity in Table 2).

**Table 2:** Differentiation of IDH-mutated and IDH-wildtype gliomas: classification results for the independent test data, calculated as the mean value from 100 repetitions (100 runs)

Performance metric	LDA	Lasso	RF	GBM	XGBoost	Nnet
AUC	0.787 [0.497:1.000]	0.807 [0.563:1.000]	0.811 [0.518:0.951]	0.783 [0.514:0.946]	0.783 [0.484:0.981]	0.794 [0.510:0.973]
Accuracy	0.789 [0.500:0.971]	0.817 [0.667:0.971]	0.773 [0.611:0.915]	0.779 [0.667:0.889]	0.784 [0.667:0.944]	0.779 [0.611:0.889]
Cohen’s Kappa	0.433 [-0.141:0.923]	0.415 [-0.174:0.923]	0.329 [-0.163:0.773]	0.196 [-0.174:0.679]	0.367 [-0.174:0.852]	0.279 [-0.098:0.702]
Precision/PPV	0.889 [0.727:1.000]	0.866 [0.750:1.000]	0.857 [0.750:1.000]	0.820 [0.750:0.931]	0.867 [0.750:1.000]	0.841 [0.765:1.000]
Recall/sensitivity	0.838 [0.538:1.000]	0.911 [0.714:1.000]	0.856 [0.680:1.000]	0.927 [0.714:1.000]	0.861 [0.680:1.000]	0.892 [0.714:1.000]
AUPRC	0.918 [0.734:1.000]	0.925 [0.753:1.000]	0.937 [0.780:0.987]	0.919 [0.774:0.985]	0.915 [0.731:0.995]	0.922 [0.679:0.993]
F1-score	0.857 [0.613:0.982]	0.884 [0.769:0.982]	0.853 [0.741:0.945]	0.866 [0.785:0.933]	0.86 [0.739:0.964]	0.861 [0.741:0.933]

Feature preselection was performed using recursive feature elimination. Six different machine learning algorithms were tested for subsequent model construction. Abbreviations: LDA = linear discriminant analysis, Lasso = least absolute shrinkage and selection operator regression, RF = random forest, GBM = gradient boosting machine, NNet = neuronal network, AUC = area under the curve, PPV = positive predictive value, AUPRC = area under the precision-recall curve. Values in brackets: 95% confidence interval.

The 100-fold repetition we used, each with a new feature preselection, can theoretically result in 100 differently composed models. Therefore, the question arises how different the 100 models obtained actually are in terms of their feature composition. For each feature that was selected using RFE in at least one run, we determined the frequency with which the respective feature was selected in the total of 100 runs. Table 3 summarizes the frequencies for the 10 most often selected features. In addition, the table contains the corresponding p-values. To calculate the p-values, all features were first analyzed using the Shapiro-Wilk normality test. Normally distributed features were subsequently analyzed using Bartlett’s test for homogeneity of variances. In the case of equal variance in the two groups (IDH-mutant/IDH-wildtype), the normally distributed features were further analyzed using Student’s test. Finally, the non-normally distributed features were analyzed using the Wilcoxon test (Mann-Whitney-U-test). Assuming a significance threshold of α < 0.05, all 10 features listed in Table 3 can be regarded as significantly discriminating.

The age of the patients at the study date was selected almost without exception (in 99% of cases). The two features “shape.Flatness” and “gldm.DependenceVariance” were also selected very frequently (in 94% and 84% of all runs respectively). The following four features were included in about 2/3 of all models.

**Table 3:** Most frequently selected features for the prediction of IDH mutation status (IDH-mutant vs. IDH wildtype) using recursive feature elimination

Level of importance	Feature name	Number of runs included	*p*-value
1	age_at_study_date	99	0.000001
2	shape.Flatness	94	0.000013
3	gldm.DependenceVariance	84	0.000098
4	glrlm.ShortRunEmphasis	66	0.000027
5	shape.LeastAxisLength	66	0.000821
6	gldm.DependenceNonUniformityNormalized	65	0.000284
7	glrlm.LongRunEmphasis	63	0.000021
8	glcm.InverseVariance	56	0.000390
9	gldm.SmallDependenceEmphasis	56	0.000418
10	firstorder.Median	48	0.047786

The column “Number of runs included“ shows the frequency of selection during the 100 runs for the corresponding feature.

We were interested to know how sensitively the model results depend on the respective individual feature composition and how important the less frequently selected features are for the model performance. Therefore, we developed models with an increasing number of the features listed in Table 3. Starting with a univariate model, which contained only the patient age at the study date as a fixed feature, the features listed in Table 3 were added successively in descending order of feature importance. All models with an increasing number of included features were again optimized 100 times each based on different data partitions. The mean performance achieved was subsequently determined using the corresponding independent test data. The approach used was therefore exactly the same as before, except that this time the final 100 models did not contain different features, but fixed features. With this approach and the use of Lasso regression, which had previously proven to be the best machine learning algorithm we tested, the highest mean model performance was achieved in the case where six features (i.e. the features “age_at_study_date” to “gldm.DependenceNonUniformityNormalized“) were included in the models. These 6-feature Lasso regression models with fixed features resulted in the mean performance values presented in Table 4. Using independent test data, the model yielded a mean AUC of 0.847 [0.577:1.000], a mean accuracy of 0.831 [0.667:0.944], a mean recall/specificity of 0.911 [0.714:1.000], and a mean AUPRC of 0.945 [0.770:1.000]. The numbers in brackets indicate the 95% confidence intervals. The performance values are even slightly higher compared to the previously determined performance values (see Table 2). For the sake of clarity, Table 4 also lists again the corresponding values from Table 2 (“Lasso” column) which were determined using RFE and Lasso regression, i.e., based on different feature compositions.

**Table 4:** Prediction of IDH mutation status using the six most important features according to Table 3 (“Fixed features” column) and using different features according to the “Lasso” column in Table 2.

Performance metric	Lasso with six fixed features	Lasso using RFE and different features
AUC	0.847 [0.577:1.000]	0.807 [0.563:1.000]
Accuracy	0.831 [0.667:0.944]	0.817 [0.667:0.971]
Cohen’s kappa	0.471 [– 0.098:0.852]	0.415 [– 0.174:0.923]
Precision/PPV	0.882 [0.765:1.000]	0.866 [0.750:1.000]
Recall/sensitivity	0.911 [0.714:1.000]	0.911 [0.714:1.000]
AUPRC	0.945 [0.770:1.000]	0.925 [0.753:1.000]
F1-score	0.893 [0.769:0.966]	0.884 [0.769:0.982]

Both approaches used Lasso regression. All results were calculated based on independent test data and as mean values of 100 repetitions (100 runs). Values in brackets: 95% confidence interval.

**Fig. 3:**
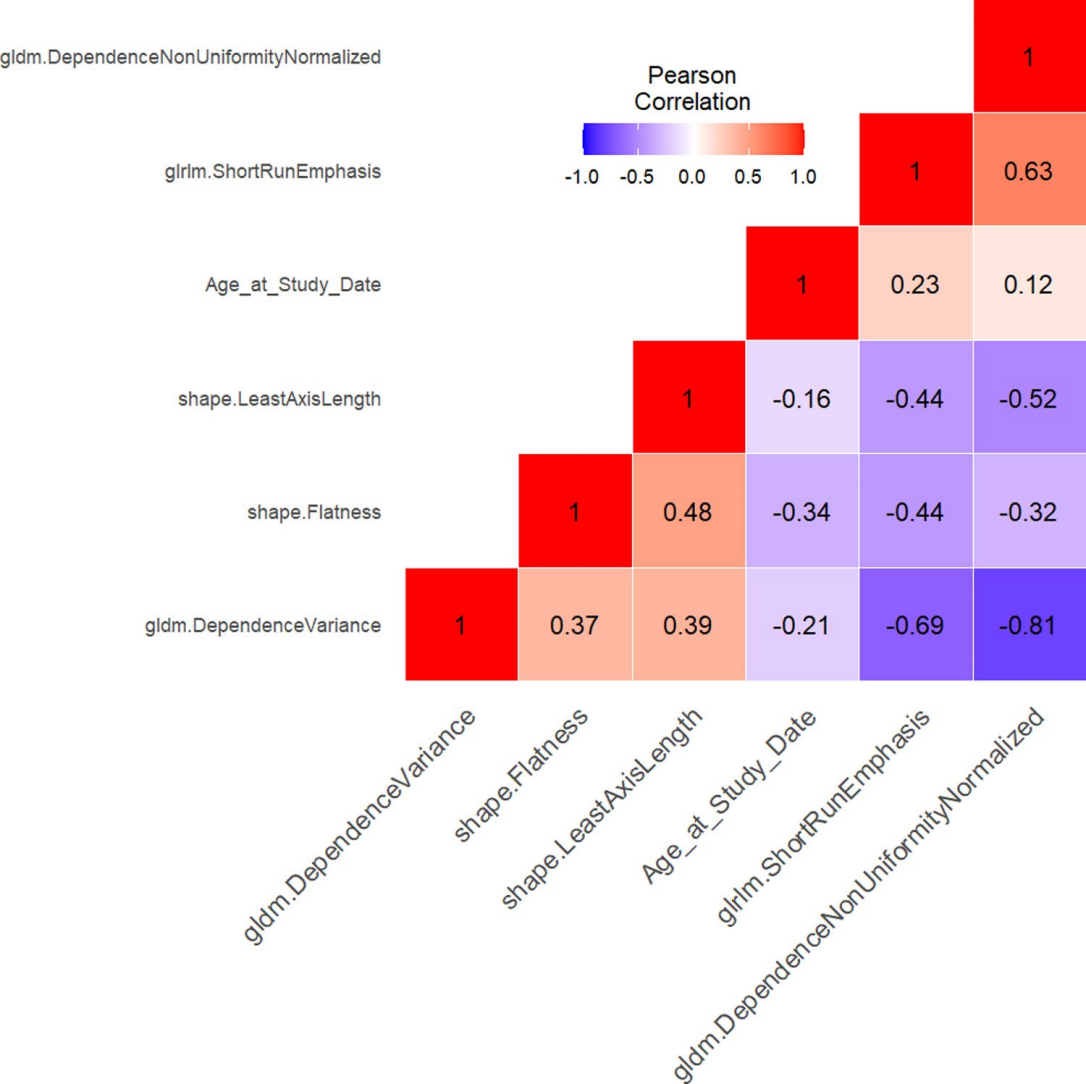
Prediction of IDH mutation status in gliomas: pearson correlation matrix of the 6 final model features.

The comparison of the two result columns in Table 4 shows, that the models with different and fixed features both have a very high and stable performance in predicting the IDH mutation status and that the previously less frequently selected features do not contribute to the model performance. The Pearson correlation matrix for the final model, which contains the six final model features in fixed form, is shown in Fig. 3. As explained, the most important model feature for the prediction of IDH mutation status (see Table 3) is the age of the patient. This feature exhibits only very low correlations with the other five model features.

Lastly, since in this study we are mainly interested in the discriminatory power that can be achieved in predicting IDH mutation status based on preoperative native CT images, we additionally computed a model containing only radiomic features. For this purpose, we used only the features 2 to 6 from Table 3 (i.e., the features “shape.Flatness” to “gldm.DependenceNonUniformityNormalized”). The age of the patient was therefore explicitly no longer included in this model. Based on this 5-feature model, again using independent test data, we obtained a mean AUC of 0.818 [0.536:0.964], a mean precision of 0.823 [0.750:0.960], a mean recall/specificity of 0.901 [0.714:1.000], and a mean AUPRC of 0.947 [0.837:0.991]. These discriminatory power values are only slightly lower than those of the corresponding model including patient age (see Table 4). The analysis shows that even when using only radiomic factors calculated based on preoperative native CT images, IDH mutation status can be predicted with high discriminatory power. This was already indicated by the low p-values of many of the radiomic features in Table 3.

## Discussion

In this study, we demonstrated that the IDH mutation status in gliomas can be predicted non-invasively and with high accuracy using radiomics and machine learning applied to preoperative native CT images. Two key highlights of our study are, firstly, that unlike many other studies which rely on MR images, our study utilizes CT images, and secondly, that the predictions are based on preoperative medical images obtained without the use of a contrast agent or radioactive tracer. Our best model, incorporating only six features, was developed using RFE and Lasso regression. On independent test data, we achieved a very high and stable performance in distinguishing IDH-mutated from IDH-wildtype gliomas, with an excellent mean AUPRC of 0.945. As noted, IDH mutation status is particularly crucial in the context of the 2021 WHO classification. IDH-wildtype gliomas are assigned to WHO grade 4. Since our study cohort only included grade II and III gliomas according to the 2016 WHO classification, we could now easily use our results to determine which of these gliomas should be reclassified as WHO grade 4 gliomas according to the 2021 WHO classification. Following these considerations, in addition to predicting IDH mutation addition, our model and the features it contains could also be used to draw conclusions about whether a glioma should potentially be reclassified with respect to the 2021 WHO classification. Our results therefore also show that our model can be used very well to determine reclassifications to be made based on the current WHO classification. Such findings can be of great importance for the further treatment of the patients.

In addition to some radiomic factors, the age of the patients proved to be particularly important for the differentiation of IDH-mutated and IDH-wildtype gliomas. In our study cohort, patients with IDH-mutated gliomas had an average age of only 42.26 years (standard deviation 10.96 years). In contrast, the average age of patients without an IDH mutation was 57.77 years (standard deviation 14.99 years). Regarding the age of the patients included in our study, there is therefore a boundary at around 50 years between patients with IDH-mutated and IDH-wildtype gliomas. This finding is consistent with the results of a study of Andrews et al.^46^. They observed higher rates of IDH-mutant gliomas in their group of patients younger than 55 years compared to their group of patients older than 55 years. The observation that older patients show higher rates of IDH-wildtype gliomas and that IDH-wildtype gliomas are in turn associated with the highest tumor grade 4 could also be part of the reason why the expected survival time of the patients depends strongly on their age^19^. On average, patients with an IDH mutation exhibit significantly longer survival times than patients with IDH-wildtype gliomas^47^.

To the best of our knowledge, there are currently very few studies investigating the prediction of IDH mutation status using radiomics and machine learning based on CT images. Zhu et al. invested the value of contrast-enhanced CT texture analysis in predicting IDH mutation status of intrahepatic cholangiocarcinoma (ICC)^48^. Using a support vector machine based on contrast-enhanced CT images, they achieved an AUC of 0.813 in their validation cohort. A second CT-based study to determine the IDH mutation status in ICCs was conducted by Idris et al.^49^. However, they used a comparatively small study cohort including only 22 patients. In contrast, as described above, there are numerous studies investigating the predictability of IDH mutation status based on MR images/ PET images. Three corresponding meta-analyses were conducted by Suh et al.^50^, Di Salle et al.^51^ and Kalaroopan et al.^52^. Due to the very heterogeneous study cohorts used in the individual sub-studies in terms of cohort size, composition of tumor grades included, imaging techniques used and many other influencing factors, it is very difficult to validly compare these results with our results based on native CT images. However, the accuracy and AUC values reported in these studies are roughly the same as those obtained in our study. In any case, based on our results, we cannot find any evidence that a non-invasive determination of IDH mutation status based on CT images is inferior to a corresponding procedure based on MR/PET images.

The determination of the IDH mutation status is often based on contrast-enhanced MR images^52–56^. However, some recent studies have shown that contrast agents can be deposited in the body^57,58^. Accordingly, there is a growing interest in avoiding the use of contrast agents as far as possible. Some studies suggest that the amount of contrast agent administered could be significantly reduced for both MR and CT imaging for various diagnostic issues^59,60^. In line with these studies, our results also suggest that the use of contrast agents could be avoided for some diagnostic questions in the future. At this point, it should be noted that it is of course also theoretically conceivable to determine the IDH mutation status completely non-invasively using native MR images instead of native CT images, as it was done in our study. Many of the common MR sequences also enable non-invasive imaging. However, it is evident that many of the corresponding studies use contrast-enhanced MR images for the prediction of IDH mutation status (see^50–52^). This may suggest that contrast agents could have a positive effect on the MR image-based prediction of IDH mutation status.Our study has some limitations. First, it is important to note its retrospective nature. Additionally, our cohort of 92 patients is relatively small, with only 22 IDH-wildtype patients included. To further enhance the discriminatory power in predicting IDH mutation status, it would be crucial to train the models with a significantly larger number of IDH-wildtype cases. Despite the methodology we used, which is based on 100 repetitions, overfitting of the models cannot be completely ruled out. It should be borne in mind that machine learning models can of course only predict characteristics that they have previously learned during training. If, for example, certain tumor subtypes were not or not sufficiently present in the study cohort used, it cannot be ruled out that a poorer performance will be achieved with corresponding cases. To assess the performance of our models and possible overfitting even more reliably, we consider it important to further validate the models based on larger study cohorts. In this context, other possible influencing factors, such as the type of CT scanner used or the energy of the X-rays used, should also be analyzed in detail. Despite these limitations, our study demonstrates that machine learning algorithms can non-invasively predict the IDH mutation status of gliomas based on preoperative native CT images. This approach has the potential to reduce the number of biopsies and expedite treatment planning.

## Conclusion

Our results demonstrate that the IDH mutation status can be predicted with high accuracy using machine learning algorithms applied to preoperative native CT images. We achieved a very high discriminatory power in predicting IDH mutations. However, because our study cohort included only a small number of IDH-wildtype gliomas, our findings should be validated with larger study cohorts and potentially in prospective studies.

## Data Availability

The datasets used and/or analysed during the current study available from the corresponding author on reasonable request.
